# Pre-therapy fasting slows epithelial turnover and modulates the microbiota but fails to mitigate methotrexate-induced gastrointestinal mucositis

**DOI:** 10.1080/19490976.2020.1809332

**Published:** 2020-08-26

**Authors:** H. R. Wardill, A. R. da Silva Ferreira, S. Lichtenberg Cloo, R. Havinga, H. J. M. Harmsen, W. P. Vermeij, W. J. E. Tissing

**Affiliations:** a Adelaide Medical School, The University of Adelaide, Adelaide, Australia; b Department of Pediatrics, University Medical Centre Groningen, The University of Groningen, Groningen, The Netherlands; c Department of Medical Microbiology, University Medical Centre Groningen, The University of Groningen, Groningen, The Netherlands; d Princess Máxima Center for Pediatric Oncology, Utrecht, The Netherlands; e Oncode Institute, Utrecht, The Netherlands

**Keywords:** Mucositis, methotrexate, gastrointestinal toxicity, dietary restriction, acute fasting, microbiota, microbiome, citrulline

## Abstract

**Background:**

Recent findings by Tang et al. (2020) show dietary restriction (30%, 2 weeks) prevents methotrexate-induced mortality by modulation of the microbiota, specifically the expansion of *Lactobacillus*. While fundamentally insightful, upscaling this schedule is a major obstacle to clinical uptake. Here, we evaluate a safe and clinically achievable schedule of pre-therapy fasting for 48 h on microbiota composition, body composition and intestinal proliferation, and assess its impact on the severity of methotrexate-induced gastrointestinal mucositis using a validated preclinical rat model.

**Methods:**

Age- and weight-matched male Wistar rats were treated with a sublethal dose of 45 mg/kg methotrexate with or without pre-therapy fasting. The impact of acute fasting on epithelial proliferation, body composition and the microbiota was assessed using plasma citrulline, Ki67 immunohistochemistry, miniSpec and 16S rRNA sequencing. The severity of gastrointestinal mucositis was evaluated using plasma citrulline and body weight.

**Results:**

Whilst pre-therapy fasting slowed epithelial proliferation and increased microbial diversity and richness, it also induced significant weight loss and was unable to attenuate the severity of mucositis in both age- and weight-matched groups. In contrast to Tang et al., we saw no expansion of *Lactobacillus* following acute fasting.

**Conclusions:**

Our findings suggest that the beneficial effects of acute fasting are masked by the detrimental effects on body weight and composition and lacking influence on *Lactobacillus*. Future studies should consider alternative fasting schedules or aim to induce comparable microbial and mucosal manipulation without compromising body composition using clinically feasible methods of dietary or microbial intervention.

## Introduction

Methotrexate (MTX) is a dihydrofolate reductase inhibitor capable of inducing cell cycle arrest and irreversible DNA damage in highly proliferative malignant cells.^1^ While a common chemotherapeutic agent used for the treatment of acute leukemia and a variety of solid tumors, its use is limited due to various dose-limiting toxicities, namely bone marrow suppression and mucositis.^2^

Mucositis affecting the gastrointestinal tract (GI-M) is estimated to occur in 60–100% of patients, with severe, potentially lethal symptoms requiring intensive supportive care and prompting dose reductions or treatment discontinuation.^3^ As such, GI-M has profound impacts on the likelihood of remission, the psychosocial wellbeing of the patient and is associated with a profound economic burden.^4,5^ Despite the clear need, there remains no gold standard method to prevent MTX-induced mucositis.^6^

The most recent advance in our understanding of MTX-induced mucositis has been the impact of the microbiota,^7,8^ in particular the microbial composition at the time of MTX administration. Preliminary evidence from our laboratory shows that antibiotic-induced disruption of the microbiota worsens MTX-induced mucositis, increasing MTX-mortality and impairing mucosal recovery. These results align with existing preclinical findings^9^ and clinical risk factors, with antibiotic use prior to chemotherapy associated with increased GI-M severity.^10^ Similarly, while probiotics have shown some benefits in mitigating the severity of MTX associated GI-M,^11^ translational success is variable.^12^ These findings have prompted an enthusiastic investigation of how the microbiota can be best modulated to improve MTX outcomes.

We read with interest the recent work by Tang et al., (2020) demonstrating that dietary restriction (DR; 30%) protects against MTX-induced mortality via modulation of the microbiota.^9^ While fundamentally insightful, these findings are translationally challenging, with long-term DR unlikely to be feasible in most oncology settings. This is particularly pertinent when considering the DR schedule employed by Tang and colleagues which commenced 2 weeks prior to MTX administration. When scaling up to human equivalent schedules, this would require long-term DR to be implemented thus impacting the ability to deliver first-line therapy in a timely fashion. Here, we show comparable microbial modulation and slowed epithelial proliferation following a clinically feasible 48 h fasting and evaluated its impact on MTX-induced gastrointestinal mucositis.

## Methods

### Ethical statement and husbandry specifications

All experiments were conducted in accordance with the ethical guidelines approved by the Dutch de Centrale Commissie Dierproeven (CCD) and the Institutional Animal Care and Use Committee of the University Medical Center Groningen, University of Groningen (RUG), under the license number 15338–01. All animals were individually housed in conventional, open cages at the Centrale Dienst Profdieren (CDP; Central Animal Facility) at the University Medical Center Groningen. Rats were housed under 12 h light/dark cycles with *ad libitum* access to autoclaved AIN93 G rodent chow (unless fasting) and sterile water. Sawdust bedding was provided in all cases as well as a toilet roll for enrichment. All cages were randomly arranged across racks to prevent potential bias.

### Preclinical model of MTX-induced GI-M and fasting conditions

The impact of fasting on MTX-induced GI-M was assessed using a validated preclinical model as previously described.^13,14^ Briefly, male Wistar rats (150–180 g, N = 9/group) were treated with a single dose of MTX (45 mg/kg, 50 mg/ml; obtained from Pharmachemie Holding B.V. The Netherlands) or a volume-equivalent dose of 0.9% NaOH administered intravenously via the penile vein under anesthetic (3% isoflurane) on d 0. Rats randomized to the acute fasting groups were given *ad libitum* access to water; however, all food was removed for 48 h prior to MTX treatment. Rats were single housed to enable accurate fecal collection and assessment of dietary intake. Food and water intake were measured daily by manually weighing the contents of the chow hopper and water bottle. During the experimental period, HRW was responsible for maintaining animal water and chow eliminating the possibility of animal caretakers to change water bottles or refresh chow.

Acute fasting was performed in age- and weight-matched groups. Rats were sacrificed 10 d post-MTX treatment via isoflurane anesthesia, cardiac puncture and cervical dislocation. An exploratory cohort of animals (N = 3 age-matched fasted, N = 3 fed) were sacrificed on d 0 to evaluate the impact of acute fasting on intestinal architecture. At sacrifice, the intestine was resected, flushed with ice-cold 1 X phosphate-buffered saline (PBS, pH 7.4) and was drop-fixed in 10% neutral-buffered formalin.

### Plasma citrulline

The primary outcome of the study was plasma citrulline, an indicator of small intestinal enterocyte mass and a validated biomarker of small intestinal GI-M.^15^ Repeated blood samples (75 μl) were collected from the tail vein into EDTA-treated hematocrit capillary tubes on d −2 (baseline), 0 (day of MTX), 2, 4, 6 and 10. Citrulline was determined in 30 μl of plasma (isolated from whole blood via centrifugation at 4000 g for 10 min) using automated ion-exchange column chromatography as previously described.^16^ The change in plasma citrulline from d −2 to 0 was used to determine the impact of acute fasting on epithelial proliferation. Citrulline analysis from d 0–10 was used as a biomarker of GI-M.

### Body composition

To determine the impact of acute fasting on body composition, rats were weighed daily from the initiation day of fasting to d 10. On d −2 (baseline) and d 0, body composition was also assessed using the Bruker MiniSpec LF90 as per manufacturer’s guidelines.

### Histology and immunohistochemistry

In a pilot cohort of exploratory male rats (N = 3 fed, N = 3 fasted), routine hematoxylin and eosin (H&E) staining was performed to evaluate intestinal architecture. Briefly, drop-fixed jejunal tissue was processed and embedded into paraffin wax. Four-micrometer sections were cut on a rotary microtome and mounted onto glass Superfrost® slides. H&E staining was performed as per routine protocols and slides were scanned using the Hamamatsu Photonics Digital Slide Scanner (NanoZoomer S60). Images were evaluated using the NDP.view2 software. Villus height and crypt depth were measured using annotation tools in NDP.view2. Ten well-oriented crypts/villi were measured per slide and an average calculated per animal.

To validate plasma citrulline as a marker of epithelial proliferation, we also evaluated the proliferative capacity of the small intestinal post-fasting using Ki67 immunostaining. Briefly, 4 μm sections of jejunum were cut on a rotary microtome and mounted onto kp+ slides. After drying, sections were dewaxed in xylene and rehydrated through graded ethanol. Antigen retrieval was performed using 800 ml of pre-heated 10 mM citrate buffer (pH 6.0). Slides were immersed and microwaved for 15 min before being cooled for 30 min at room temperature. Endogenous peroxidase activity was quenched with 1% H2O2 in methanol before sections were blocked in 10% normal goat serum. Sections were incubated with the primary antibody (rabbit anti-Ki67 (Fisher Scientific, RM9106-s0) 1:50 with 10% normal goat serum in 1 X PBS) overnight in a humid chamber at 4°C. After three washes in 1 X PBS/Tween20, the secondary antibody was applied (Vector goat anti-rabbit/biotin BA-1000, 1:250 with 1 X PBS) before being incubated with the ABC/PO-complex (1:25 with 1 X PBS, Vector, PK-4000). Antibody binding was visualized with DAB substrate and tissue was counterstained in hematoxylin before being dehydrated and cleared in graded ethanols and xylene. Sections were coverslipped and scanned using the Hamamatsu Photonics Digital Slide Scanner (NanoZoomer S60). Positive Ki67 cells were counted per crypt side for 10 crypts per slide and an average calculated per animal.

### Microbiota analysis

Microbiota composition was assessed using 16S rRNA sequencing on paired fecal samples collected pre- and post-fasting. DNA was extracted using the double bead-beater procedure from Yu and Morrison (2004)^17^ and QIAamp DNA Stool Minikit guidelines (Qiagen). DNA was quantified using the NanoDrop UV Visible Light Spectrophotometer and the V3-V4 region amplified using polymerase chain reaction (PCR). Each PCR reaction contained 1 μl of 10 μM 341 f forward primer (V3F: 5ʹaatgatacggcgaccaccgagatct 3ʹ), 25 μl Phire HS II Master Mix, 22 μl DNase free water, 1 μl of 10 μM 806r barcoded reverse primer and 1 μl DNA template (100 ng/μl). These were denatured at 98°C for 30 sec and amplified over 31 cycles of 98°C for 5 sec, 50°C for 5 sec, 72°C for 10 sec. Samples were held at 72°C for 1 min and kept at 4°C until collection. Amplification was confirmed using gel electrophoresis. Size selection and fragment removal were performed using AMPure XP beads as per manufacturer’s guidelines before the final PCR products were normalized to 2 mM and pooled to form a single library which was stored at −4°C until sequencing.

Sequencing was performed using the MiSeq Benchtop Next Generation Sequencer (Illumina). The paired-end sequencing data received from Illumina software were processed by the software PANDAseq (version 2.5)^18^ and QIIME (version 1.7.0).^19^ Readouts with a quality score below 0.9 were discarded by PANDAseq to increase the quality of the sequence readouts. De novo OTU-picking was performed without chimera filtering with Greengenes (version 13.5) as a reference database. Data were visualized and analyzed using the Qiagen CLC Genomics Workbench 12 (kindly provided by Qiagen to Wardill HR).

### Statistical analyses

All data were analyzed using GraphPad Prism v8.0. Data were analyzed for normality using the D’Agostino-Pearson Normality test, and when confirmed were analyzed using a two-way analysis of variance (ANOVA). For repeated measures, data were analyzed using a mixed model with Geisser’s Greenhouse correction. For correlations, a simple linear regression with Pearson correlation coefficients was used to determine the strength and significance. *P* < .05 was considered significant. All statistical analyses are described in figure legends.

## Results

### Acute fasting slows epithelial turnover but severely depletes fat stores

**Figure 1.**
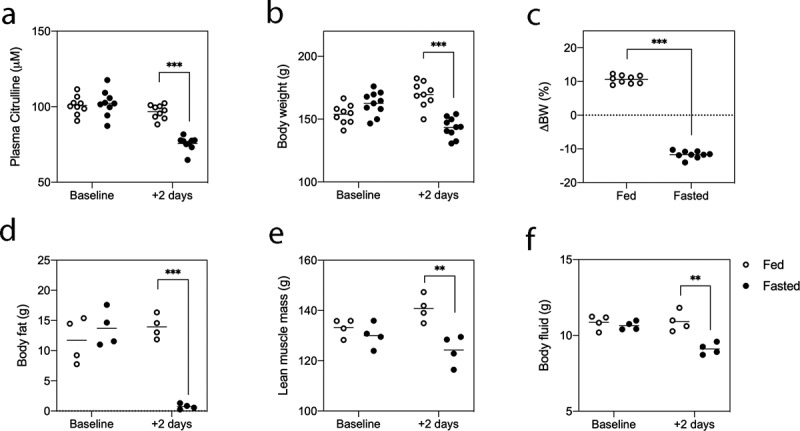
Acute fasting slows epithelial turnover and induces significant changes in body composition. Rats were given ad libitum access to food and water (fed), or water only (fasted) for 48 h. Plasma citrulline (a), body weight (B/C) and body composition (D/E/F) were assessed at baseline and following the fasting period. Data points shown for all rats with N = 4/group for body composition analysis and N = 9/group for all other analyses. Data were assessed using a two-way ANOVA. P < .05 was considered statistically significant.

To evaluate the impact of acute fasting on epithelial proliferation, plasma citrulline was assessed pre- and post-fasting (Figure 1a). Plasma citrulline is a biomarker of small intestinal enterocyte mass with decreases occurring in response to slowed turnover or epithelial injury. Acute fasting for 48 h caused a significant decrease in plasma citrulline (−25.43 ± 3.01%, ***P < .0001, Figure 1a). Control animals showed no significant change in plasma citrulline over the 48-h period. Fasting also caused significant weight loss (−11.79 ± 0.34%, ***P < .0001, Figure 1b,c), and almost complete depletion of body fat (13.95 ± 1.98 g [baseline] to 0.74 ± 0.44 g [after fasting], ***P < .0001, Figure 1d). This was paired with a decrease in lean muscle mass (**P = .0011, Figure 1e) and body fluid (**P = .0003, figure 1f). Despite the change in body fluid, water intake did not significantly differ between groups during the fasting period.

**Figure 2.**
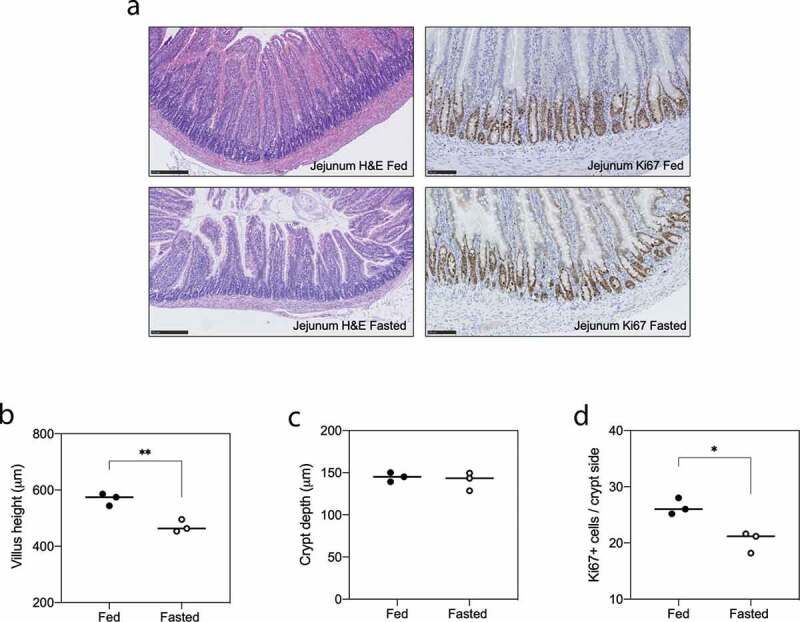
Acute fasting induces villus atrophy and reduced proliferation in the jejunum. Intestinal architecture and proliferation were assessed using H&E staining and Ki67 immunohistochemistry, respectively, in N = 6 animals. Fasting decreases villus length (**P = .0054; A/B) yet had no effect on crypt depth (c). The number of Ki67+ cells in the jejunum decreased following 48-h fasting (*P = .01; D). Data shown as individual points and mean. All data were analyzed with an unpaired t-test, where *P* < .05 was considered significant.

Histological analysis of the jejunum indicated mild but significant villus blunting in the jejunum (567.9 ± 12.6 μm vs 470.5 ± 12.6 μm, **P = .0054, Figure 2a,b). No changes were seen in crypt depth (Figure 2c). The impact of fasting on epithelial turnover was confirmed with Ki67 immunostaining, with a significant decrease in Ki67+ cells after fasting (*P = .01, Figure 2).

### Acute fasting modulates the microbiota

**Figure 3.**
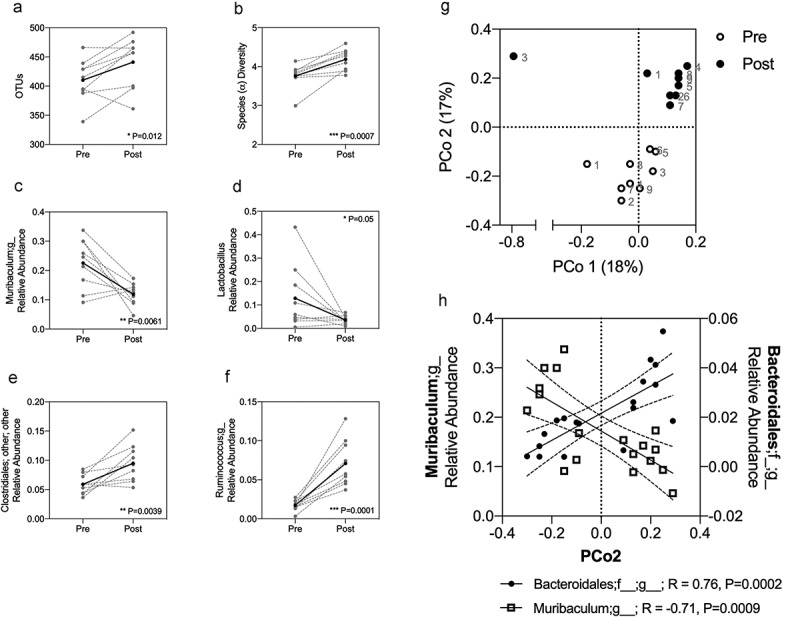
Acute fasting promotes microbial diversity and richness and induces compositional changes in the fecal microbiota. Microbiota composition was assessed in fasted rats, with paired fecal samples collected pre- and post-fasting. Increased richness, indicted by OTUs (a), and alpha diversity (b) were observed after fasting. Significant changes in the relative abundance of *Muribaculum* (c), *Lactobacillus* (d), *Clostridiales* (e) and *Ruminococcus* (f) were identified. Compositional changes aligning with PCo2 were identified post-fasting (g), correlating with *Muribaculum* and *Bacteroidales* (h). A-F show paired results from individual animals (gray hashed lines), with the mean depicted by a solid black line. A-F were analyzed using a paired t-test where *P* < .05 was significant. A simple linear regression with Pearson correlation coefficients were used in H to determine strength and significance.

The effect of acute fasting on microbiota composition was assessed in N = 9 fasted male rats using 16S rRNA sequencing. Stool samples were collected at baseline and after 48 h of fasting. Acute fasting increased microbial richness and alpha diversity, indicated by the number of operational taxonomic units (OTUs, Figure 3a) and Shannon’s index (Figure 3b). Significant changes were observed in the relative abundance of Muribaculum;g_ (Figure 3c, **P = .0061), *Lactobacillus* (Figure 3d, *P = .05), Clostridiales (Figure 3e, **P = .0039) and Ruminococcus;g_ (figure 3f, ***P = .0001). Fasting induced a compositional shift in the microbiota aligning with Principle Component (PCo)2 (Figure 3g), with strong correlations observed between PCo2 and Muribaculum;g_ (***P = .0009, Figure 3h) and Bacteroidales (***P = .0002, Figure 3h). For all statistically significant correlations with PCo2, please see Table 1.

**Table 1.** Taxonomic classifications significantly correlated with PCo2.

	r	P	R^2^
k__Bacteria;p__Bacteroidetes;c__Bacteroidia;o__Bacteroidales;f__;g__	0.7617	0.0002	0.5802
k__Bacteria;p__Proteobacteria;c__Gammaproteobacteria;o__Enterobacteriales;f__Enterobacteriaceae;g__	−0.7626	0.0002	0.5815
k__Bacteria;p__Bacteroidetes;c__Bacteroidia;o__Bacteroidales;f__[Odoribacteraceae];g__Odoribacter	0.7527	0.0003	0.5666
k__Bacteria;p__Firmicutes;c__Bacilli;o__Lactobacillales;f__Streptococcaceae;g__Lactococcus	−0.7521	0.0003	0.5656
k__Bacteria;p__Firmicutes;c__Bacilli;Other;Other;Other	−0.7225	0.0007	0.5219
k__Bacteria;p__Bacteroidetes;c__Bacteroidia;o__Bacteroidales;f__S24-7;g__	−0.7143	0.0009	0.5102
k__Bacteria;p__Firmicutes;c__Bacilli;o__Lactobacillales;Other;Other	−0.7106	0.0009	0.505
k__Bacteria;p__Firmicutes;c__Clostridia;o__Clostridiales;f__Lachnospiraceae;Other	−0.6957	0.0013	0.484
k__Bacteria;p__Firmicutes;c__Clostridia;o__Clostridiales;f__Ruminococcaceae;g__	0.6835	0.0018	0.4672
k__Bacteria;p__Firmicutes;Other;Other;Other;Other	−0.6669	0.0025	0.4448
k__Bacteria;p__Firmicutes;c__Bacilli;o__Lactobacillales;f__Streptococcaceae;Other	−0.6065	0.0076	0.3678
k__Bacteria;p__Firmicutes;c__Bacilli;o__Turicibacterales;f__Turicibacteraceae;g__Turicibacter	−0.5905	0.0099	0.3487
k__Bacteria;p__Firmicutes;c__Clostridia;Other;Other;Other	−0.5709	0.0133	0.3259
k__Bacteria;p__Proteobacteria;c__Deltaproteobacteria;o__Desulfovibrionales;f__Desulfovibrionaceae;g__Bilophila	0.5298	0.0238	0.2806
k__Bacteria;p__Actinobacteria;c__Actinobacteria;o__Actinomycetales;f__Micrococcaceae;g__Rothia	−0.5258	0.025	0.2765
k__Bacteria;p__Firmicutes;c__Bacilli;o__Lactobacillales;f__Lactobacillaceae;Other	−0.5188	0.0274	0.2692
k__Bacteria;p__Firmicutes;c__Erysipelotrichi;o__Erysipelotrichales;f__Erysipelotrichaceae;g__Allobaculum	0.513	0.0295	0.2632
k__Bacteria;p__Proteobacteria;c__Deltaproteobacteria;o__Desulfovibrionales;f__Desulfovibrionaceae;g__Desulfovibrio	−0.512	0.0299	0.2621
k__Bacteria;p__Firmicutes;c__Clostridia;o__Clostridiales;f__Ruminococcaceae;Other	0.5098	0.0307	0.2599
k__Bacteria;p__Firmicutes;c__Clostridia;o__Clostridiales;f__Lachnospiraceae;g__Dorea	0.5009	0.0342	0.2509
k__Bacteria;p__Actinobacteria;c__Actinobacteria;o__Actinomycetales;f__Micrococcaceae;Other	−0.4914	0.0384	0.2414
k__Bacteria;p__Firmicutes;c__Bacilli;o__Lactobacillales;f__Carnobacteriaceae;g__Granulicatella	−0.4886	0.0397	0.2387
k__Bacteria;p__Firmicutes;c__Clostridia;o__Clostridiales;Other;Other	0.4874	0.0402	0.2376
k__Bacteria;p__Bacteroidetes;c__Bacteroidia;o__Bacteroidales;f__Rikenellaceae;g__	0.4848	0.0414	0.235
k__Bacteria;p__Bacteroidetes;c__Bacteroidia;o__Bacteroidales;f__[Odoribacteraceae];g__Butyricimonas	0.4744	0.0467	0.2251
k__Bacteria;p__Firmicutes;c__Clostridia;o__Clostridiales;f__Clostridiaceae;g__Clostridium	0.4734	0.0472	0.2241
k__Bacteria;p__Firmicutes;c__Clostridia;o__Clostridiales;f__Ruminococcaceae;g__Ruminococcus	0.4735	0.0472	0.2242

### The severity of MTX-induced mucositis is not influenced by pre-therapy fasting

**Figure 4.**
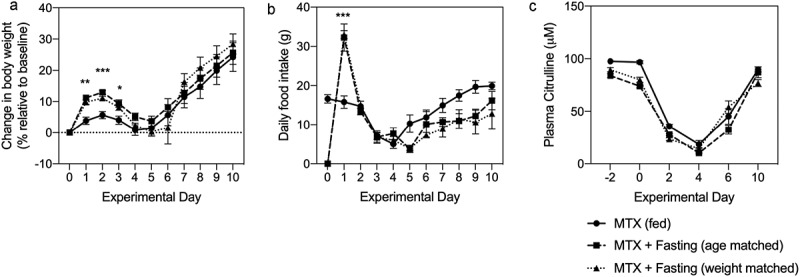
MTX-induced gastrointestinal mucositis is not influenced by acute fasting. Rats (N = 9/group) were treated with a single dose of MTX (45 mg/kg) and gastrointestinal mucositis determined by relative body weight (a), food intake (b) and plasma citrulline (c). Significant increases in relative body weight were identified in age- and weight-matched rats following fasting, however this did not affect body weight during peak mucositis (d 4) or the degree of mucosal injury indicated by citrulline. Data are shown as mean±SEM and were analyzed using a mixed-effects model with Geisser-Greenhouse correction. *P* < .05 was considered significant.

We next assessed the impact of pre-therapy fasting on MTX-induced gastrointestinal mucositis (GI-M) using a validated rat model of moderate, self-limiting mucosal injury. Our primary outcome measure was plasma citrulline assessed longitudinally over 10 d. It is well documented that body compositions, in particular body mass index (BMI) and surface area, are predictors of GI-M. Given the profound weight loss induced by acute fasting, and its potential as a co-founder, we evaluated the impact of acute fasting in age- and weight-matched animals (i.e. fed and fasted rats were comparable body weight at the time of MTX). Pre-therapy fasting was unable to prevent the severity of GI-M as determined by plasma citrulline and body weight loss (Figure 4). Body weight was significantly higher in fasted animals in the first 3-d post-MTX treatment (Figure 4a, **P = .002, ***P = .0002, *P = .023). Food intake was also increased in fasted rats on the first day following MTX treatment (***P < .0001, Figure 4b). Despite this initial effect, body weight in peak GI-M (D 4) did not differ between fed and fasted animals. Similarly, the severity of the mucosal injury was unchanged throughout the time course of MTX-induced GI-M (Figure 4c).

## Discussion

Methotrexate (MTX)-induced mucositis remains a significant and unmet need that is only set to grow as the number of cancer diagnoses increase. Given the increasing recognition of its contribution to deadly treatment complications^20-22^ and late effects in survivors of childhood cancer,^23^ efforts to prevent acute mucosal injury are paramount. Here, we report microbial benefits following a clinically feasible fasting schedule, with paralleled decreases in epithelial proliferation. While encouraging, pre-therapy fasting for 48 h also caused detrimental effects on body composition and was unable to mitigate MTX-induced GI-M in a validated preclinical model.

Acute fasting has a number of benefits when compared to dietary restriction, namely its safety and feasibility in a clinical oncology setting, which has already been confirmed.^24^ When considering the challenges in scaling up a 2-week dietary restriction schedule in mice to humans, methods that exploit or build upon the insight gained from Tang et al., (2020) are critically valuable, particularly given the magnitude of protection achieved by dietary restriction in a lethal model of MTX toxicity. Previous research has shown short-term fasting (24 and 48 h) is able to protect against a lethal dose of etoposide;^25,26^ however, the mechanisms responsible were not addressed in detail. Surprisingly, despite mice losing over 20% of their body weight following fasting, survival was significantly higher compared to *ad libitum* fed controls and did not compromise the anti-tumor efficacy of etoposide. These findings challenge our conclusion that despite the observed benefits of acute fasting on the microbiota and epithelial turnover, the drastic changes in body weight, and in particular fat stores, mask these protective mechanisms. In line with findings reported by Raffaghello et al., (2008), fasted rats in the current study did recover to their pre-fast body weight very rapidly and, in fact, were significantly heavier than their fed counterparts on d 1, 2, 3 post-MTX. However, likely due to the long lag time of MTX, this was not maintained, and the degree of mucosal injury (determined by citrulline) was unchanged. This highlights the importance of using an objective biomarker of mucosal injury in addition to translatable endpoints (e.g. body weight) to provide clear insight into the degree of mucositis. It also highlights a limitation of our study, with only direct mucosal injury (citrulline) and body weight assessed in our model, both of which are affected by fasting. As such, the impact of fasting on unconfounded inflammatory markers of GI-M is unclear.

In an attempt to avoid the confounding impact on body weight (induced by fasting) at the time of MTX treatment, we also assessed the efficacy of fasting in a weight-matched cohort. Fasting in age-matched rats results in a considerable discrepancy in body weight at d 0 (time of MTX administration). Experience in our group suggests that, despite body weight-dependent dosing, reduced body weight at the time of MTX can worsen toxicity outcomes, aligning with low body mass index as a predictor of mucositis.^27^ Weight-matched rats were allowed to gain additional weight during acclimatization, to ensure that they were a comparable weight to control (fed) rats after 48 h of fasting. Contrary to our hypothesis, this did not affect the ability of pre-therapy fasting to prevent MTX-induced GI-M, indicating that it is more likely that the lack of fat stores masks the protective cellular and microbial mechanisms induced by fasting. Despite the contradictory findings to those published by Tang et al., (2020) and Raffaghello et al., (2008), there are a number of differences that must be acknowledged. Firstly, both studies reporting positive effects of fasting/dietary modulation used lethal doses of chemotherapy administered to mice. In contrast, we used a model of moderate, self-limiting mucosal injury that is closer to clinical treatment settings. As such, the mechanisms responsible for promoting survival may differ from those that govern mucosal injury. Similarly, while Raffaghello et al., (2008) report on etoposide which has a different mechanism and kinetic of cytotoxicity. Likewise, the model of MTX administration used by Tang et al. differs from our protocol (intraperitoneal administration). While common in preclinical practice, intraperitoneal administration does not align with clinical administration of MTX, and as such, the translatability of our results (where MTX is administered intravenously) is arguably stronger.

Mechanistically, however, the insight gained from Tang et al., (2020) is of interest, with antibiotic depletion of the microbiota-exacerbating MTX-mortality. This aligns with the increasing recognition of how the microbiota may regulate anti-cancer efficacy and toxicity.^7,28^ While the exact microbial changes induced by dietary restriction and fasting differ, both are associated with “beneficial” or “protective” changes in the microbiota (i.e. increased diversity/richness), emphasizing the importance of understanding how host–microbe interactions can be best exploited to optimize the outcomes of cancer therapy and minimize toxicity. However, a major difference in the microbial changes observed between the two studies was the abundance of *Lactobacillus*, with Tang et al., (2020) reporting a significant increase following DR (prior to MTX), which correlated with survival outcomes. This contrasts our finding of decreased Lactobacillus following acute fasting and may explain the differences observed in study outcomes. We suggest that unlike DR, acute fasting entirely depletes protein and sugar provision for resident microbes (including *Lactobacillus*) that rely on dietary sources of nutrition.^29^ This is in contrast to microbes that increased following acute fasting (e.g. *Ruminococcus*) that primarily utilize endogenous/host nutrient sources such as mucin.^30,31^ Despite these discrepancies, it is clear that manipulating the microbiota in a manner that does not detrimentally affect body composition is likely to be a fundamental step in better managing the complications of cancer treatment. It would be of particular interest if novel approaches could also target epithelial proliferation to decrease the sensitivity of intestinal stem cells to cytotoxic injury. Whether this is achieved directly (i.e. through different fasting regimens) or indirectly (i.e. via the microbiota) remains to be answered.

In summary, our study highlights that a clinically feasible schedule of acute fasting is able to induce “preferable” changes in the microbiota while decreasing the proliferative capacity of the intestinal mucosa. Whilst aligning with findings from other fasting and dietary restriction schedules, our intervention was unable to protect against MTX-induced GI-M, highlighting the challenges in modulating host–microbe interactions in a translationally meaningful manner. Future studies should focus on identifying a dietary protocol that is safe and feasible, and investigate schedules of fasting that provide greater protection in the acute phases of injury.
